# Hypophosphatasia Presenting with Pyridoxine-Responsive Seizures, Hypercalcemia, and Pseudotumor Cerebri: Case Report

**DOI:** 10.4274/jcrpe.473

**Published:** 2012-03-08

**Authors:** Hüseyin Demirbilek, Yasemin Alanay, Ayfer Alikaşifoğlu, Meral Topçu, Etienne Mornet, Alev Özön, Nurgün Kandemir

**Affiliations:** 1 Hacettepe University Faculty of Medicine, Department of Pediatrics Endocrinology, Ankara, Turkey; 2 Hacettepe University Faculty of Medicine, Department of Genetics, Ankara, Turkey; 3 Hacettepe University Faculty of Medicine, Department of Pediatric Neurology, Ankara, Turkey; 4 Université de Versailles Saint Quentin en Yvelines, France; +90 312 305 11 24+90 312 312 18 09dr_huseyin@hotmail.com

**Keywords:** Hypophosphatasia, pyridoxine-responsive seizures, bisphosphonates, Alkaline phosphatase, bone resorption, hypercalcemia

## Abstract

Hypophosphatasia (HPP) is an inborn error of metabolism characterized by defective bone mineralization caused by a deficiency in alkaline phosphatase (ALP) activity due to mutations in the tissue-nonspecific ALP (TNALP) gene. The clinical expression of the disease is variable. Six forms of HPP are identified according to age at presentation and clinical features. Patients with the infantile form are normal at birth. First symptoms appear within the first 6 months of life. Along with skeletal findings, HPP patients may present with hypercalcemia, seizures, pseudotumor cerebri, and pulmonary insufficiency. Seizures in HPP are refractory to conventional antiepileptic drugs, but are responsive to pyridoxine. Herein, we report a case of HPP who presented with pyridoxine-responsive seizures in the early neonatal period and was found to have hypercalcemia, skeletal demineralization and increased intracranial pressure. Key words: Hypophosphatasia, pyridoxine-responsive seizures, bisphosphonates, alkaline phosphatase, bone resorption, hypercalcemia

**Conflict of interest:**None declared.

## INTRODUCTION

Hypophosphatasia (HPP) is a rare inherited disorder characterized by defective bone and tooth mineralization, together with low serum and bone alkaline phosphatase (ALP) activity (1). The incidence of the severe form is estimated to be 1/100 000. However, mild forms may be more common (2,3). The symptoms are highly variable in their clinical expression, which range from stillbirth with no mineralized bone to premature teeth loss alone (1). Six forms of the disease are identified according to age at diagnosis and severity of symptoms. These clinical forms are: perinatal lethal, prenatal benign, infantile, childhood, adult forms, and odontohypophosphatasia. The transmission of severe forms is autosomal recessive, while milder forms may be transmitted as dominant or recessive autosomal traits (3). The diagnosis is based on clinical and radiological findings of bone demineralization in the face of a low serum ALP level. Molecular analysis of the tissue-nonspecific (i.e. liver/bone/kidney) ALP (TNALP) gene can verify the genetic defect (4).

Patients with the infantile form may appear normal at birth; however, clinical signs appear within the first 6 months of life (3). Patients may present with respiratory complications due to rachitic deformities of the chest, premature craniosynostosis that may result in increased intracranial pressure (IICP), hypercalcemia and its symptoms such as irritability, poor feeding, anorexia, vomiting, polydypsia, polyuria, dehydration, constipation, hypotonia and, less commonly, seizures. 

Here, we present the clinical and biochemical findings as well as the molecular analysis of a case of infantile HPP associated with hypercalcemia, pyridoxine-responsive seizures (West syndrome) and pseudotumor cerebri.

## CASE REPORT

A 4-month-old boy was admitted to our clinic for hypercalcemia and seizures refractory to treatment. He was the fifth child of parents who were first cousins. He was born at 36 weeks’ gestation after an uneventful pregnancy. Delivery was spontaneous via the vaginal route. His birth weight was 3000 g. He was discharged from the hospital on the second postnatal day, but was readmitted to the same hospital on the next day with hypotonia, poor feeding, and generalized tonic-clonic seizures. The patient’s electroencephalogram (EEG) revealed epileptic activity in the left frontal area. He was started on phenobarbital (PB) and phenytoin (DPH), which controlled his tonic-clonic seizures. However, the myoclonic convulsions as well as bouts of crying and screaming continued. An EEG taken at age 2 months showed hypsarrhythmia. Synacthen and valproic acid were started, but 2 days later, the baby developed involuntary movements of the extremities and was found to have hypercalcemia. At this time, DPH and valproic acid as well as synacthen were discontinued, and in the following days, the patient received only PB. Later, clonazepam was added to the treatment regimen, but the involuntary movements of the extremities continued until 4 months of age when he was referred to our center.

**Figure 1:**
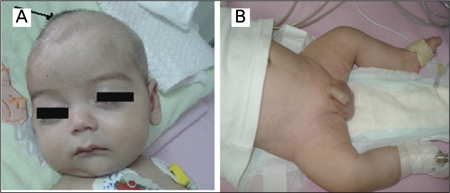
(A) bulging of anterior fontanelle, and (B) hypotonic, short andbowed extremities of the patient

**Figure 2(A,B,C,D):**
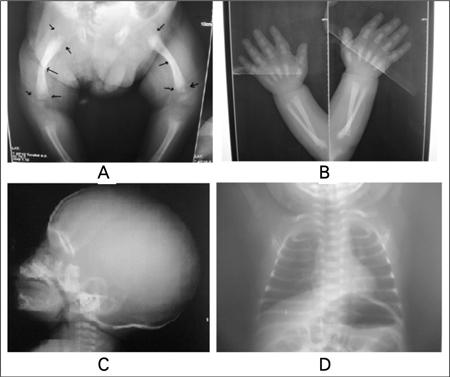
X-rays show bowing and angulation in the long bones,defective mineralization in the epiphyses and metaphyses, demineralizationin the skull, and a normal chest X-ray

Physical examination at the time of admission revealed short stature (length 55 cm, <3^rd^ percentile). Weight was also below the 3rd percentile for age (4400 g). Generalised muscle hypotonia, a bulging anterior fontanelle, short and bowed lower extremities (Figure 1), and a simian crease in the left palm were noted. There were no physical signs of rachitic changes in the chest. X-rays showed mineralization defect in the long bones, cupping of the metaphyses, and defective ossification in the skull bones (Figure 2A,B,C). The chest X-rays showed no abnormalities (Figure 2D). Blood chemistry findings were: total calcium 14.6 mg/dL (N:8.5-10.5); ionized calcium 1.6 mmol/L (N: 1.14-1.29); phosphorus 4.81 mg/dL (N:3.5-5.5); ALP 7 IU/L (N: 150-240), parathyroid hormone (PTH) <3 pg/mL (N:12-95), 25-hydroxyvitamin D [25(OH)D] 32.7 ng/mL (N:10-60), magnesium 1.68 mg/dL (N: 1.4-1.9). Repeat ALP level was also remarkably low (11 IU/L) suggesting HPP when considered together with the clinical and radiological findings of mineralization defect. Urinary calcium/creatinine ratio was 1.37. Urinary phosphoethanolamine excretion was elevated (172.4 μmol/L; N:0-10) supporting the diagnosis of HPP. Other markers of bone turnover were also elevated (urinary deoxypyridinoline 157.4 nMDPD/mMcre, N:2.3-5.4; serum osteocalcine 64.7 ng/mL, N:9.9-27). Serum electrolytes, glucose, blood gases, hepatic and renal function tests as well as routine urinalysis were all within normal limits. Renal ultrasonography revealed medullary hyperechogenicity suggesting medullary nephrocalcinosis. Cranial magnetic resonance (MR) imaging showed mild ventricular dilatation. Further laboratory investigation directed to the etiology of seizures included tandem mass spectrometry, urinary and plasma amino acid analysis, urinary organic acid analysis, visual evoked potential (VEP) and brain auditory evoked potential (BAEP) studies, as well as electroretinography (ERG), and all were within normal limits. Intravenous (IV) hydration with saline plus furosemide was started at the time of admission to treat hypercalcemia, and dietary calcium intake was restricted. This therapy had minimal effect on calcium levels and serum calcium was 13.8 mg/dL on the 3rd hospital day. The hypercalcemia was resistant to other treatment options including prednisolone (2 mg/kg/d) and calcitonin (10 U/kg/d). Normocalcemia was achieved with a single dose of 0.5 mg/kg IV pamidronate.

A lumbar puncture (LP) was performed to investigate the cause of the bulging anterior fontanelle. Opening pressure was increased (270 cm H2O). Cerebrospinal fluid (CSF) glucose and protein levels were all within normal limits, and cultures were negative. Absence of any organic lesion on MR imaging as well as the normal CSF findings in the face of IICP suggested pseudotumor cerebri. No additional therapy was recommended since the patient did not have symptoms of IICP such as vomiting or irritability. A follow-up LP was also not performed since there was no change in the patient’s clinical condition suggestive of either resolution or progression of pseudotumor cerebri.

The patient was on PB and clonazepam at the time of admission, but his seizures (involuntary twitching) continued despite this treatment. Pyridoxine was tried on the second day after his admission when a diagnosis of HPP was considered. Seizures refractory to previous antiepileptic therapy resolved completely following pyridoxine administration (100 mg/day initial and 50 mg/day maintenance dose, PO). A follow-up EEG, carried out 6 days after the initial dose of pyridoxine, showed voltage suppression but no epileptiform anomaly. The patient was discharged after resolution of hypercalcemia and seizures. Further molecular analysis performed at the S.E.S.E.P laboratory in France confirmed the diagnosis of HPP. A homozygous c.659G>C (p.G220A) missense mutation was found on exon 7. The parents were heterozygous for the mutation.

The patient was lost to follow-up after discharge. He was followed by a local university close to his residence. Unfortunately, we learned that he had died at the age of 8 months in the course of pulmonary infection.

## DISCUSSION

HPP is a rare disorder among the various causes of hypercalcemia. On the other hand, hypercalcemia is frequent in HPP patients, particularly in those with the infantile form of the disease (5,6,7). Hypercalcemia is thought to be due to impaired bone mineralization in the presence of normal bone resorption. TNALP deficiency impairs bone mineralization by inhibition of the growth of hydroxyapatite crystals caused by increased inorganic pyrophosphate (PPi) which is a substrate for the defective enzyme. 

There is some controversy in the literature about the preferred treatment for hypercalcemia in patients with HPP. Dietary restriction of calcium intake is recommended in most papers. On the other hand, there is much conflict about the use or harmful effects of saline solution, furosemide, chlorothiazide, glucocorticoids, calcitonin and bisphosphonates (pamidronate) (5,6,7). The conflict arises from a number of reasons. Firstly, since this is a rare disorder, information about the treatment and effect of the treatment on hypercalcemia is based on reports of single cases. Secondly, the disease has a tendency to improve with age even in the severe forms, thus, it is not unusual that a report suggests sudden resolution of hypercalcemia which may coincide with some form of conventional treatment for hypocalcemia. Thirdly, probably there is some individual variation with regard to bone formation and resorption among these patients. Osteoclasts are generally accepted to function normally and lead to age-appropriate bone resorption in patients with HPP. In parallel with this information, markers of bone resorption (i.e. pyridinoline, deoxypyridinoline) are within normal limits in most case reports, however, there are also patients in whom bone resorption is increased, as demonstrated by the presence of the same markers, which would be expected to influence the response to treatment. All in all, therapies directed at improving hypercalcemia are expected to worsen bone demineralization. Bisphosphonates are not expected to be effective in treating hypercalcemia in patients with HPP since bone resorption is normal in this condition. Despite this knowledge, since other treatments had not been effective to normalize serum calcium and also because urinary deoxypyridinoline was elevated, suggesting increased resorption, a single dose of pamidronate was tried in our patient and was found to successfully decrease the serum calcium level to the high-normal range. We also found that this effect could be sustained by continuing glucocorticoid therapy and by calcium restriction. Unfortunately, the patient succumbed because of pulmonary infection at the age of 8 months.

Pyridoxine-responsive seizures have been reported in association with HPP, and in fact, they are considered to be an indicator of disease severity (8,9,10,11). Several specific mutations have been suggested to be responsible (8,9,10,11,12). A very similar case has been reported by Baumgartner-Sigl et al. previously (8). That case presented with pyridoxine-responsive seizures as a neonate and developed skeletal demineralization, hypercalcemia, hypercalciuria and nephrocalcinosis consistent with HPP later in infancy.The mechanism of pyridoxine-responsive seizures in HPP is explained by defective metabolism of pyridoxal 5-phosphate (PLP), which is the phosphorylated form of pyridoxine (vitamin B6). TNALP hydrolyses PLP, and the unphosphorylated pyridoxine crosses the blood-brain barrier to be re-generated as PLP (13). PLP is a co-enzyme for glutamic acid decarboxylase, which plays a crucial role in the synthesis of gamma-aminobutyric acid (GABA) (10). The inability of defective TNALP to cleave PLP may result in vitamin B6 deficiency, thus reducing the levels of inhibitory neurotransmitter GABA in the brain leading to seizures (14). Studies of ALP activity in the primate brain support the important role of this enzyme in neurotransmission. Kinetic characterization of TNALP mutations showed that some mutations were efficient in hydrolysing PPi but inefficient in hydrolysing PLP, which could explain why some patients manifest more severe seizures than others (15,16,17).

In the current case, we observed IICP as well, initially suggested by a bulging fontanelle and later documented by an elevated CSF pressure. MR imaging excluded organic lesions as the cause of IICP, suggesting pseudotumor cerebri. Pseudotumor cerebri may arise from abnormalities in serum calcium levels in hypo- or hypercalcemia (18). Teber et al (19) previously reported a case of HPP who had pseudotumor cerebri which resolved after glucocorticoid therapy. They suggested that, as found in their patient, pseudotumor cerebri could be ascribed to hypercalcemia and resolved after correction of the hypercalcemia with glucocorticoid therapy.

Alternatively, premature closure of sutures is held responsible for IICP in patients with HPP. Craniosynostosis is a well-known feature of the infantile and childhood HPP. Collmann et al (20) reported a series of 7 children among 20 cases of HPP who had craniosynostosis and discussed the functional problems arising from HPP. In our patient, cranial sutures and anterior fontanelle were wide open, thus, craniosynostosis cannot be held responsible for the IICP.

The molecular defect detected in our case was previously reported in 2 cases with severe perinatal HPP (21). Interestingly, in one of those cases, the patient was homozygous for the mutation c.659G>C and the parents were first cousins of Turkish origin. In the second case, the patient was a compound heterozygote with c.659G>C and another defect, and the parent carrying the c.659G>C mutation was again of Turkish origin. These findings strongly suggest a founder effect of this mutation in Turkey. The mutation affects an amino acid that is not located in the functional 3D domain of the TNALP protein. However, the residue G220 is highly conserved during evolution and another mutation affecting the same residue (c.659G>T, p.G220V) is also responsible for HPP (22), corroborating the disease-causing role of the mutation.

In conclusion, HPP is a rare cause of hypercalcemia in childhood and should be suspected when serum ALP is found to be low. In some cases, HPP may be associated with pyridoxine-responsive seizures. Pseudotumor cerebri may also develop in some cases. The current patient had all of these clinical features. In addition, it is noteworthy that the mutation described in this patient was shown in 2 other non-related Turkish families as well, a finding which suggests a founder effect of this mutation in Turkey.
